# General Exploitation Theory

**DOI:** 10.3389/fpsyg.2026.1938950

**Published:** 2026-10-13

**Authors:** Norbert Meskó

**Affiliations:** Department of Cognitive and Evolutionary Psychology, Faculty of Humanities and Social Sciences, Institute of Psychology, University of Pécs, Pécs, Hungary

**Keywords:** contextual recalibration, exploitation, exploitative behavior, psychological appraisal, social context, social decision-making

## Abstract

Exploitation is a feature of human social life, yet its study remains fragmented across psychological, economic, philosophical, and social-scientific traditions. We propose General Exploitation Theory (GET), an integrative framework that separates the conceptual question of what exploitation is from the causal question of what makes exploitative action more or less likely. GET defines exploitation as a relational process in which an actor seeks or obtains self-benefit by using a feature of another party’s relational position to secure a position-dependent advantage. Such an advantage is an incremental benefit or more favorable terms that would be reduced or unavailable in a comparable interaction in which the underlying contribution, resource, or value remained the same but the enabling positional feature was absent or neutralized. The causal architecture distinguishes objective exploitability from exploitation-relevant appraisal and proposes four appraisal dimensions: expected actor benefit, anticipated self-cost, perceived appropriability, and target-welfare weighting. Relational and social-contextual conditions create or modify objective exploitability and, together with individual characteristics, calibrate exploitation-relevant appraisal without constituting exploitation themselves. Exploitative action is distinguished from successful exploitative outcomes, while behavioral responses and their outcomes can modify subsequent appraisals, relational conditions, and institutional environments through recursive feedback. GET treats interpersonal, organizational, and digital exploitation as context-specific instantiations of an architecture rather than as phenomena requiring separate explanatory models. The framework generates falsifiable predictions concerning appraisal mediation, functional equivalence, contextual recalibration, divergent responses to exploitability, and recursive feedback. GET thereby provides a psychologically explicit framework for explaining how exploitable relational conditions can become exploitative action.

## 1 Introduction

Exploitation is a recurrent feature of human social life and a central concept across multiple disciplines, including psychology, economics, political science, philosophy, and sociology (Roemer, 1982; Rusch et al., 2026; Wertheimer, 1999). Yet these traditions have approached exploitation from substantially different perspectives. Some emphasize unfair advantage, coercive conditions, or the terms on which one party benefits from another (Mayer, 2007; Wertheimer, 1999), others focus on structural inequalities that enable some actors to benefit systematically at the expense of others (Acemoglu and Robinson, 2012; Roemer, 1982; Sidanius and Pratto, 1999), and still others examine individual differences associated with manipulative, self-serving, or antagonistic behavior (Moshagen et al., 2018). This diversity has generated a rich literature but has also left exploitation conceptually fragmented, with different traditions often using the same term for partly different relational phenomena (Rusch et al., 2026; Wertheimer, 1999).

One source of this fragmentation is the coexistence of normative and functional approaches. Normative accounts ask when an interaction should be regarded as exploitative, often emphasizing unfairness, coercion, reciprocity, or the terms under which one party benefits from another (Mayer, 2007; Walzer, 1983; Wertheimer, 1999). By contrast, a functional or behavioral perspective shifts attention to the processes through which actors pursue benefits, respond to opportunities, and impose costs on others. These perspectives need not be competing, but they address different theoretical problems. For the purposes of theory building, it is therefore useful to distinguish two analytically separate questions: what makes a relational process exploitative, and what makes exploitative behavior more or less likely to occur? The first concerns the conceptual boundaries of exploitation; the second concerns its causal explanation. Conflating these questions risks treating the features used to identify exploitation as if they were empirical predictors of its occurrence.

A second problem concerns levels of explanation. Research on individual differences has linked antagonistic personality characteristics, low Honesty–Humility, and related dispositions to manipulative and self-serving behavior (Ashton and Lee, 2007; Moshagen et al., 2018; Muris et al., 2017). In parallel, social and institutional research has documented how power differences, resource dependencies, information asymmetries, and institutional arrangements can create opportunities for actors to appropriate benefits while shifting costs to others (Acemoglu and Robinson, 2012; Piketty, 2020; Rosenblat and Stark, 2016; Wood et al., 2019). These literatures illuminate different parts of the same problem, but the pathways through which individual-level psychological processes interact with relational and social-institutional conditions have received less systematic theoretical integration (Rusch et al., 2026). A general account of exploitation therefore requires more than identifying exploitative dispositions or exploitative environments separately; it requires specifying how characteristics of actors and contexts jointly shape exploitative action and how the consequences of such actions can, in turn, modify the conditions under which subsequent interactions occur.

General Exploitation Theory (GET) is proposed as an integrative framework for addressing these problems. It proceeds in two steps. First, GET specifies exploitation as a relational phenomenon and distinguishes it from adjacent forms of self-interest, competition, cheating, deception, coercion, and opportunism. Second, it addresses the separate causal question of how exploitable relational conditions are psychologically appraised and translated—or not translated—into exploitative action. At this causal level, GET connects individual-level psychological processes with the relational and social-contextual conditions that create, amplify, or constrain opportunities for exploitation. The framework is explicitly recursive: exploitative actions and their consequences may alter both actors’ subsequent appraisals and the relational or institutional conditions governing future interactions. Thus, GET treats exploitation neither as the direct expression of a stable exploitative disposition nor as the deterministic consequence of an unequal environment, but as a context-sensitive process emerging from their structured interaction.

The present article develops this framework in five stages. First, it provides a minimal relational definition of exploitation and clarifies its conceptual boundaries, including its relationship to adjacent constructs and difficult boundary cases. Second, it specifies the causal architecture through which objective exploitability, exploitation-relevant appraisal, individual-level calibration, and social-contextual constraints are linked to exploitative action and outcomes. Third, the framework is illustrated across a limited set of interpersonal, institutional, and digital contexts, prioritizing depth over breadth and using these contexts to demonstrate how a common causal architecture can be instantiated through different concrete mechanisms. Fourth, GET is positioned relative to existing theoretical frameworks in order to clarify what is gained by integrating psychological and social-contextual processes within a recursive explanatory architecture. Finally, the framework derives a limited set of distinctive and falsifiable empirical predictions, distinguishing implications that follow specifically from GET from empirical regularities already established in its constituent literatures. By separating conceptual definition from causal explanation while retaining a cross-level and context-sensitive systems perspective, GET aims to provide a more precise and empirically tractable framework for understanding exploitation across human social contexts.

## 2 What is exploitation? Conceptual definition and boundaries

### 2.1 A minimal relational definition of exploitation

Exploitation has been conceptualized in different ways across philosophical, economic, and social-scientific traditions, but a recurrent feature of these accounts is that exploitation is relational: one party benefits, or seeks to benefit, through some feature of another party’s position (Mayer, 2007; Roemer, 1982; Wertheimer, 1999). Philosophical accounts have commonly emphasized taking unfair advantage of another party, including cases in which the interaction is voluntary or mutually beneficial (Mayer, 2007; Wertheimer, 1999), whereas economic approaches have emphasized how one party’s gains may depend on resources, contributions, or positions held by others (Roemer, 1982). GET adopts this relational core while separating the question of what exploitation is from the question of what makes it more likely to occur.

This definition is proposed as a theoretical construct definition within GET rather than as an empirical claim about the criteria by which lay observers identify exploitation. How people actually judge and categorize exploitation remains a separate empirical question. Recent experimental evidence indicates that both power asymmetry and inequality systematically increase judgments that an interaction is exploitative (Ferguson et al., 2026). GET also does not define exploitation by reference to a universal standard of fair exchange, equal outcomes, or balanced reciprocity. Although such standards may be relevant to the moral evaluation of exploitation, making them definitional would require an independent account of what constitutes a fair allocation across qualitatively different relational contexts.

GET defines exploitation as a relational process in which an actor seeks or obtains self-benefit by using a feature of another party’s relational position to secure a position-dependent advantage. A position-dependent advantage is an incremental benefit or more favorable terms that arise because the relevant feature of the other party’s position enables the actor to shift the allocation or appropriation of benefits, costs, resources, contributions, or other value in the actor’s favor. The critical distinction is therefore between benefiting from what another party contributes to an interaction and obtaining additional advantage from a feature of that party’s relational position. The former characterizes many ordinary forms of cooperation, exchange, and competition and is not sufficient for exploitation. Exploitation requires the latter.

*The relevant comparison is counterfactual*. A position-dependent advantage exists when the actor’s advantage would be smaller, or the terms available to the actor less favorable, in a comparable interaction in which the underlying resource, contribution, or value associated with the other party remained substantially the same but the particular feature of that party’s relational position enabling the additional advantage was absent or neutralized. The comparison therefore concerns the source of the actor’s incremental advantage rather than whether the observed outcome departs from an idealized standard of fairness. Features capable of creating such an advantage may include limited alternatives, dependency, informational disadvantage, weak bargaining capacity, or high costs of resistance, although no particular feature is necessary for exploitation.

*Consider employment*. An employer may benefit from an employee’s labor without exploiting the employee. The employee’s contribution is instrumentally necessary for the employer’s benefit, but dependence of benefit on another party’s contribution is not itself exploitation. If, however, the employer uses the employee’s severely limited alternatives or dependency to obtain more favorable terms than would be available for a comparable contribution in the absence of that positional feature, the resulting increment constitutes a position-dependent advantage. The same distinction applies more generally to exchange: unequal outcomes and successful bargaining do not by themselves establish exploitation, whereas a distinct feature of another party’s relational position may provide a route through which the actor secures an additional advantage.

This counterfactual criterion does not require the exploited party to experience an absolute net loss. A transaction may improve the position of both parties while still allowing one party to obtain a position-dependent advantage, a possibility long recognized in philosophical analyses of mutually beneficial exploitation (Mayer, 2007; Wertheimer, 1999). A worker with severely limited alternatives, for example, may prefer accepting employment to remaining unemployed while the employer simultaneously uses those limited alternatives to secure more favorable terms. The relevant comparison for identifying exploitation is therefore not necessarily the absence of the transaction. It is the comparable interaction in which the positional feature generating the actor’s additional advantage is absent or neutralized.

Conversely, unequal outcomes, differential benefits, dependency, vulnerability, or asymmetry are not by themselves sufficient to establish exploitation. These conditions become relevant when they provide a route through which one party can obtain a position-dependent advantage from another party’s relational position. This distinction allows GET to retain a minimal relational definition while treating the specific conditions that create exploitability, the psychological processes through which actors recognize and evaluate those conditions, and the constraints affecting whether they act on them as separate components of the causal explanation developed below.

### 2.2 What exploitation does not require

The minimal definition proposed above deliberately separates the relational structure of exploitation from conditions that may make exploitation more likely. This distinction is particularly important for asymmetry and constraint, both of which were treated as defining components in the original formulation of GET. Differences in power, information, dependency, resources, or capacity to resist can create favorable conditions for exploitation, but a pre-existing asymmetry is not conceptually necessary. An exploitable advantage may instead emerge within an interaction—for example, when deception creates an informational disadvantage that did not previously exist. Conversely, substantial asymmetries can persist without being exploited. The same reasoning applies to constraints. Monitoring, accountability, sanctions, reputational consequences, and opportunities for resistance can affect whether an exploitable situation is acted upon (Fehr and Gächter, 2002; Jiao et al., 2021; Tyler, 2006), but strong constraints do not make exploitation conceptually impossible, just as weak constraints do not make it inevitable. Asymmetry and constraint are therefore treated in GET as causal and contextual conditions rather than criteria by which exploitation is defined.

Exploitation likewise does not require violation of a social or institutional norm. Norms regulate what communities regard as permissible, expected, or legitimate, whereas exploitation concerns the relational process through which one party obtains advantage through another’s position. The two can coincide, but neither entails the other. A prohibited behavior may violate a norm without exploiting another party, while an exploitative arrangement may be normalized, institutionally permitted, or socially accepted (Acemoglu and Robinson, 2012; Jost, 2020). Treating norm violation as a defining feature would therefore make the classification of exploitation dependent on prevailing social standards and would obscure precisely those cases in which exploitative practices become embedded in established relationships or institutions.

Nor is the absence of consent a necessary condition for exploitation. Philosophical analyses have long recognized the possibility of voluntary and mutually beneficial exploitation: a party may knowingly accept an interaction because it improves their situation while the other party nevertheless takes advantage of features of their position to obtain unusually favorable terms (Mayer, 2007; Wertheimer, 1999). Consent is therefore relevant to the interpretation of an interaction but does not by itself determine whether that interaction is exploitative. Within GET, the evidential meaning of consent therefore depends on such considerations as information, available alternatives, dependency, and the practical capacity to refuse or negotiate. In conceptual terms, consent concerns acceptance of an interaction or its terms; exploitation concerns how one party obtains advantage through the other’s position.

For the same reason, exploitation does not require the target to suffer an absolute net loss. A party may receive a benefit and still be exploited if the actor’s advantage is obtained through the relational process specified above (Mayer, 2007; Wertheimer, 1999). Conversely, harm, inequality, dependency, or weak protection alone is insufficient to establish exploitation. These conditions may increase objective exploitability, alter bargaining positions, or affect the consequences of an interaction, but they do not by themselves establish the position-dependent advantage required by the definition.

### 2.3 Distinguishing exploitation from adjacent constructs

A central challenge for a general theory of exploitation is to avoid conceptual overreach. If exploitation is defined too broadly, it risks becoming indistinguishable from self-interest, opportunism, cheating, deception, coercion, or other forms of behavior through which actors pursue advantages. If defined too narrowly, however, it may fail to capture the relational structure shared by otherwise different exploitative interactions. The relevant constructs should therefore not be treated as mutually exclusive categories distinguished by a common checklist of features. Rather, they describe different properties of behavior or interaction and can consequently intersect within the same event. Table 1 summarizes these distinctions by identifying the primary theoretical focus of each construct and its relationship to exploitation.

**Table 1:** Conceptual distinctions between exploitation and adjacent constructs.

Construct	Primary theoretical focus	Identifying feature	Relation to exploitation
Self-interest	Actor’s motivational orientation	Pursuit of outcomes that benefit the self	Self-interest may motivate exploitation but does not specify how benefit is obtained and is therefore neither sufficient nor distinctive of exploitation.
Opportunism	Strategic pursuit of self-interest	Self-interest seeking through guile, including concealment, distortion, deception, or circumvention	Opportunistic behavior may be exploitative when the actor uses a feature of another party’s relational position to obtain a position-dependent advantage, but opportunism and exploitation are not conceptually identical.
Cheating	Rules and cooperative expectations	Violation or circumvention of rules or expected cooperation to obtain an advantage	Cheating may create or facilitate an exploitative advantage, but it can occur without exploitation, and exploitation need not involve cheating.
Deception	Information and belief	Deliberate attempt to create or maintain a belief the actor regards as false or misleading	Deception can create an informational advantage that enables exploitation, but it is neither necessary nor sufficient for exploitation.
Coercion	Control over another party’s choice or action	Restriction of another’s effective choice through force, threat, or comparable compulsion	Coercion can be used as a mechanism of exploitation, but coercion can occur without exploitation, and exploitation may be entirely non-coercive.
Exploitation	Relational advantage-taking	Self-benefit is pursued or obtained through a position-dependent advantage enabled by another party’s relational position. The advantage would be reduced or unavailable if, with the underlying contribution, resource, performance, or value held substantially constant, the enabling positional feature were absent or neutralized.	Focal construct of GET; it may intersect with the other constructs but is not reducible to any of them.

The broadest distinction is between exploitation and self-interest. Self-interest concerns what an actor seeks: outcomes that advance the actor’s own welfare or goals. Exploitation, by contrast, concerns how an advantage is pursued or obtained in relation to another party. The distinction therefore separates motivational orientation from relational structure rather than treating exploitation as an unusually strong form of self-interest.

Opportunism is more closely related to exploitation because it concerns the strategic pursuit of advantage. In Williamson's (1985) formulation, opportunism involves self-interest seeking with guile and may include concealment, distortion, or other strategic attempts to secure favorable outcomes. Nevertheless, opportunism and exploitation are not synonymous. Opportunistic behavior becomes exploitative in the sense specified by GET when the actor uses a feature of another party’s relational position to obtain a position-dependent advantage. Conversely, strategic advantage-seeking can occur without altering the terms, allocation, or appropriation of value through such a positional feature. Exploitation therefore represents neither a synonym nor a simple subtype of opportunism; the constructs identify different features that may co-occur within the same behavior.

Cheating and deception likewise describe more specific properties of behavior. Cheating concerns the violation or circumvention of rules or cooperative expectations in order to obtain an advantage, whereas deception concerns the deliberate manipulation of another party’s beliefs or information (Abe, 2011; Gneezy, 2005; Shalvi et al., 2015). Either can facilitate exploitation, but neither defines it. Cheating or deception becomes relevant to exploitation when it creates or enables a position-dependent advantage—for example, when informational manipulation allows an actor to secure terms or appropriate value that would not be available if the relevant informational disadvantage were absent. Conversely, exploitation can occur without either cheating or deception, as when an actor openly uses another party’s dependency or limited alternatives to secure additional advantage. Deception also illustrates why pre-existing asymmetry is not necessary for exploitation: strategic manipulation of information can itself create the relational feature through which a position-dependent advantage subsequently becomes available.

Coercion differs again because its primary theoretical focus is control over another party’s effective choice or action. Force, threats, or comparable forms of compulsion can restrict effective choice and can be used to obtain benefits from another person (Crossman and Hardesty, 2018; Stark, 2007), thereby serving as mechanisms of exploitation in some interactions. Yet coercion is neither necessary nor sufficient for exploitation. Coercive action can occur for purposes unrelated to obtaining a position-dependent advantage from the coerced party, while exploitative interactions can remain formally voluntary and involve no threat or force (Mayer, 2007; Wertheimer, 1999). Coercion can also create exploitability rather than merely act upon a pre-existing vulnerability, because threats or force can generate the restricted alternatives or high costs of resistance through which additional advantage becomes available.

These distinctions change how conceptual overlap should be interpreted. Cheating, deception, coercion, and opportunism can provide mechanisms through which an actor obtains a position-dependent advantage, but each can also occur without doing so. The relevant question for GET is therefore not which behavioral category an action belongs to, but whether the interaction acquires the relational structure specified in Section 2.1. This distinction preserves the conceptual boundaries of exploitation while allowing the behavioral mechanisms through which it is enacted to vary across contexts.

### 2.4 Boundary cases

The usefulness of a minimal definition depends partly on how it handles cases that resemble exploitation without necessarily satisfying its relational criterion. Three boundary cases are particularly informative: legitimate exchange and regulated competition, negative externalities, and so-called self-exploitation.

*Legitimate exchange and regulated competition*. Not every interaction in which parties receive unequal benefits is exploitative. Market exchange, bargaining, employment, and competitive interactions routinely produce unequal outcomes, and voluntary or mutually beneficial transactions cannot be classified as non-exploitative solely on those grounds because such transactions can themselves be exploitative (Mayer, 2007; Wertheimer, 1999). The relevant distinction is whether an actor benefits from the contributions, resources, performance, or strategic behavior that constitute the underlying interaction, or instead obtains a position-dependent advantage through a distinct feature of the other party’s relational position.

*This distinction also applies to regulated competition*. Success does not become exploitative merely because one competitor identifies and strategically responds to another’s strengths or weaknesses. Such performance characteristics are among the constitutive inputs of the competitive interaction itself. A tennis player who repeatedly targets an opponent’s weaker stroke therefore gains a strategic advantage but does not, on that basis alone, exploit the opponent in GET’s sense. By contrast, exploitation becomes possible when an actor uses a relational feature distinct from those constitutive inputs to shift the terms or appropriation of value in the actor’s favor. Inequality of outcome, strategic advantage, and position-dependent exploitation must therefore be distinguished rather than treated as equivalent.

*Negative externalities*. An actor may obtain a benefit while imposing costs on other individuals or groups without thereby exploiting them. A firm’s profitable activity may, for example, generate pollution that harms a neighboring community. If the community’s exposure to the cost is merely a side effect of the firm’s activity, the case involves a negative externality but does not necessarily satisfy GET’s relational criterion. It becomes exploitative when a feature of the affected party’s relational position provides a route to position-dependent advantage—for example, when an actor strategically locates or maintains a harmful activity where affected communities have limited capacity to resist, exit, or obtain redress, thereby enabling the actor to externalize costs on terms that would be less favorable if those limitations were absent. The distinction is therefore between costs that accompany another party’s benefit and costs whose allocation is shifted through a feature of the affected party’s relational position.

*Self-exploitation*. The relational definition also places a boundary around what is sometimes described as self-exploitation. Behaviors such as excessive work, self-neglect, or costly short-term decision making may impose substantial costs on the same individual who pursues the corresponding benefit. Such patterns may be psychologically important, but they do not constitute exploitation in the core sense used by GET because the required inter-agent relational structure is absent. This does not mean that apparently self-imposed costs are irrelevant to exploitation. An individual may, for example, overwork within an organizational arrangement that benefits from and reinforces such behavior. In such cases, the relevant question for GET is not whether the individual exploits the self, but whether another actor or institutional arrangement uses a feature of the individual’s relational position to obtain a position-dependent advantage. GET therefore restricts its core explanatory domain to exploitation between distinguishable actors or parties rather than extending the construct to intrapersonal conflict.

## 3 The causal architecture of General Exploitation Theory

### 3.1 Relational conditions and objective exploitability

The causal problem begins with a distinction between conditions that make exploitation possible and the behavior that follows from them. Relational and social-contextual conditions can create situations in which one party can potentially obtain a position-dependent advantage from another. Differences in access to resources, information, alternatives, bargaining capacity, authority, or resistance can contribute to such situations. These conditions are causally important, but they do not themselves constitute exploitation.

GET therefore distinguishes objective exploitability from exploitative behavior. Objective exploitability refers to properties of a relational situation that make a position-dependent advantage available: they permit one party to obtain an incremental benefit or more favorable terms through a feature of another party’s relational position, beyond the benefit available from that party’s underlying contribution, resource, performance, or value alone. Limited alternatives, informational disadvantage, dependency, weak bargaining position, or high costs of resistance can all contribute to objective exploitability. No particular condition is necessary, and such conditions constitute exploitability only insofar as they provide a potential route to position-dependent advantage. An actor may overlook these conditions, misinterpret them, or recognize them accurately while choosing not to exploit them. Conversely, an actor may perceive another party as exploitable even when the anticipated position-dependent advantage cannot in fact be realized.

The proximal link between these conditions and behavior is exploitation-relevant appraisal: the actor’s representation and evaluation of information bearing on the potential pursuit of a position-dependent advantage. GET proposes that this appraisal concerns four analytically separable dimensions: expected actor benefit, anticipated self-cost, perceived appropriability, and target-welfare weighting. Individual differences and contextual conditions can influence these appraisal dimensions without becoming necessary components of exploitation themselves.

The resulting causal architecture is summarized in Figure 1. Relational and social-contextual conditions can create or modify objective exploitability and can also calibrate how exploitable conditions are appraised. Objective exploitability becomes behaviorally relevant through exploitation-relevant appraisal and the selection of a behavioral response. Individual characteristics and social-contextual conditions can calibrate this process at multiple points, while the consequences of exploitative action can feed back into subsequent appraisal, relational conditions, and broader social or institutional environments. These contemporaneous calibration effects are analytically distinct from recursive feedback: the former represent inputs operating within the current interaction, whereas the latter represent changes produced by prior interactions that become inputs to subsequent interactions.

**Figure 1:**
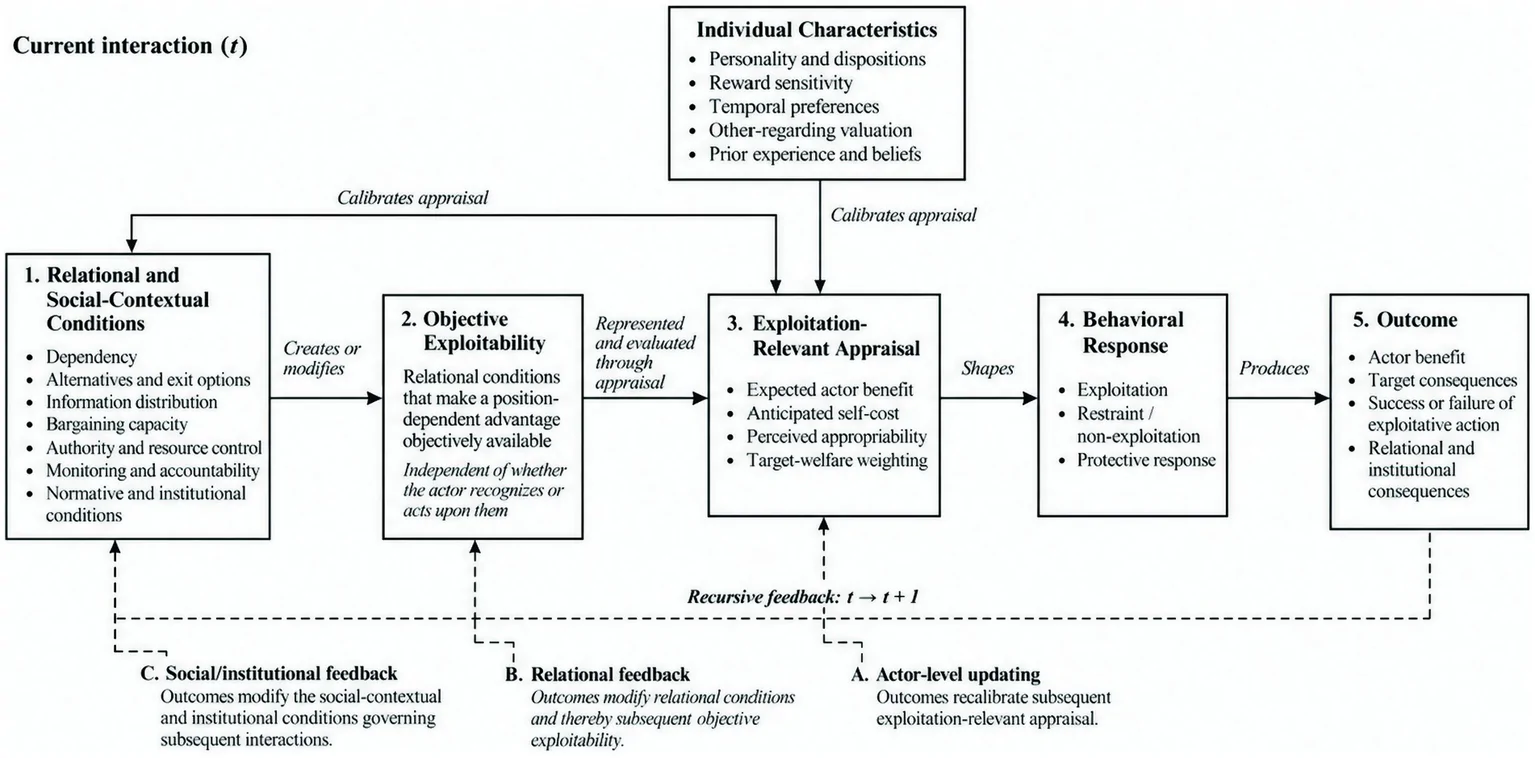
The General Exploitation Theory (GET): causal architecture and recursive feedback. Relational and social-contextual conditions can create or modify objective exploitability and can also calibrate exploitation-relevant appraisal. Objective exploitability becomes behaviorally relevant through appraisal of expected actor benefit, anticipated self-cost, perceived appropriability, and target-welfare weighting. Individual characteristics provide additional current calibration inputs to appraisal. Appraisal shapes behavioral response probabilistically, and behavioral responses generate outcomes. Outcomes can influence subsequent interactions through three temporally distinct feedback pathways: **(A)** actor-level updating recalibrates subsequent appraisal; **(B)** relational feedback modifies conditions affecting subsequent objective exploitability; and **(C)** social/institutional feedback modifies the broader contextual conditions governing subsequent interactions. Current calibration inputs operate within interaction *t*, whereas recursive feedback represents changes produced by prior interactions that become inputs at *t* + 1.

### 3.2 Exploitation-relevant appraisal

GET distinguishes four analytically separable dimensions of exploitation-relevant appraisal: expected actor benefit, anticipated self-cost, perceived appropriability, and target-welfare weighting. These dimensions identify the kinds of information relevant to pursuing an exploitative advantage; they are not proposed as necessary and sufficient components of exploitation, nor as quantities that must combine according to a fixed decision rule.

Expected actor benefit concerns the value the actor anticipates obtaining through the interaction. Benefits may include resources, status, information, labor, access, avoidance of effort, sexual or social opportunities, or other valued outcomes. What matters proximally is not the objective benefit eventually realized but the value expected by the actor at the time of appraisal. The same objectively available outcome may therefore have different motivational significance across actors or situations. Research on reward and incentive processing similarly indicates substantial individual variation in responsiveness to anticipated rewards (Berridge and Robinson, 2016).

Anticipated self-cost concerns the consequences the actor expects to incur by pursuing the advantage. These may include material penalties, detection, retaliation, reputational damage, relationship deterioration, lost future opportunities, or psychological costs associated with violating personally relevant standards. Anticipated self-cost is therefore distinct from the objective presence of external constraints. Strong formal sanctions may have little behavioral influence if detection or enforcement appears unlikely, whereas relatively weak formal regulation may matter greatly when relational or reputational consequences are salient.

Perceived appropriability concerns whether the actor represents the relational situation as permitting costs to be shifted to the target or resources, contributions, opportunities, or other value to be obtained through the target’s position. This appraisal should be distinguished from objective exploitability. Objective exploitability concerns whether the relational configuration actually permits such an advantage; perceived appropriability concerns whether the actor represents that route as usable. Dependency, informational disadvantage, limited alternatives, unequal bargaining positions, or institutional authority may create objective possibilities for advantage-taking, but these possibilities influence behavior partly through whether and how they are detected and interpreted by the actor.

The fourth dimension, target-welfare weighting, concerns the importance assigned to differences in the target’s expected welfare across the available responses. An opportunity can appear profitable, readily appropriable, and personally inexpensive while nevertheless being rejected when exploitation is expected to worsen the target’s welfare relative to available alternatives and those consequences carry sufficient weight for the actor. Target-welfare weighting is therefore not intrinsically inhibitory: its behavioral implication depends on the target’s expected welfare under exploitation relative to restraint, non-interaction, or other available responses. Evolutionary models of welfare trade-offs provide an established framework for understanding how social decision systems may assign different relative weights to one’s own welfare and that of another individual (Sell et al., 2009). GET does not equate target-welfare weighting with a welfare trade-off ratio, but WTR theory provides a useful model of how self–other valuation may vary across relationships and be recalibrated by relational information (Delton and Robertson, 2016).

The four appraisal dimensions need not covary. A potentially large benefit may also carry substantial anticipated self-cost; a highly appropriable resource may belong to a target whose welfare is strongly valued; or an actor may care little about the target while judging appropriation too costly or difficult. Similar behavioral outcomes can therefore arise through different appraisal pathways. Refraining from exploitation may reflect low expected actor benefit, high anticipated self-cost, low perceived appropriability, or—when exploitation is expected to leave the target worse off than available alternatives—strong target-welfare weighting.

Target-welfare weighting is particularly important because it is not reducible to anticipated self-cost. Concern for another party need not matter only because harming that person creates guilt or another internal penalty. The target’s welfare can enter appraisal as an independently valued outcome. Recognition of exploitability therefore need not imply exploitative motivation: under some circumstances, the same information may motivate restraint or even protection rather than appropriation.

GET does not assume that actors consciously calculate these dimensions or combine them additively, multiplicatively, or according to a common threshold. Instead, the appraisal architecture defines separable psychological dimensions whose measurement and functional integration are empirical questions. This shifts attention from asking whether an actor possesses an exploitative disposition to asking which characteristics of actors and contexts alter particular aspects of exploitation-relevant appraisal.

### 3.3 Individual-level calibration of appraisal

Individual characteristics influence exploitation in GET primarily by calibrating appraisal rather than directly producing exploitative behavior. Relatively stable dispositions and more transient psychological states can alter what actors value, how strongly they anticipate consequences, how readily they recognize appropriative possibilities, and how much weight they assign to another person’s welfare. None of these characteristics is therefore necessary or sufficient for exploitation, and the same disposition may be expressed differently across relational contexts.

Variation in reward valuation and temporal preferences can affect expected actor benefit and anticipated self-cost. Reward sensitivity alters the motivational significance of anticipated incentives, whereas temporal discounting changes the relative valuation of outcomes occurring at different points in time (Berridge and Robinson, 2016; Frederick et al., 2002). Meta-analytic evidence further links delay discounting to broader patterns of impulsive and addictive behavior, supporting its relevance to decisions involving temporally separated benefits and costs (Amlung et al., 2017). Temporal discounting is relevant to exploitation when gains and costs have different temporal distributions—for example, when an immediate gain is paired with delayed reputational or relational consequences. It is not a general exploitative mechanism, however, because exploitative benefits can themselves be delayed and relevant costs can be immediate.

Self-regulation and moral evaluation can also alter anticipated self-cost. Immediate benefits may compete with delayed reputational, relational, or other personally valued consequences, making self-regulatory processes potentially relevant to how such consequences enter appraisal. Internalized moral standards can likewise make some actions psychologically costly even when external sanctions are weak. Moral disengagement is especially relevant because it describes processes through which conduct that would otherwise conflict with moral standards can be justified or reconstructed in ways that reduce self-sanction (Bandura, 1999). In GET, moral disengagement therefore affects the internal consequences anticipated from exploitation rather than constituting an independent exploitative motive.

Other-regarding valuation bears more directly on target-welfare weighting. Empathic processes can increase the motivational importance of another person’s expected outcomes, whereas weaker concern for others can reduce the extent to which target costs counterbalance self-benefits (Lockwood et al., 2017). Welfare trade-off models provide a complementary account by proposing that the relative valuation of self and other varies across social relationships and informational contexts (Delton and Robertson, 2016; Sell et al., 2009). Empathy and welfare weighting are not assumed to be interchangeable; rather, they represent partially distinct routes through which consequences for the target may acquire motivational importance.

Individuals may also differ in their detection and interpretation of exploitability. Social-cognitive processes can affect how potentially self-serving features of a situation are interpreted and justified (Shalvi et al., 2015). Prior experience and beliefs about others may likewise shape whether dependency, informational disadvantage, limited alternatives, or other exploitable conditions are represented as usable opportunities. Such differences are especially relevant to perceived appropriability because objectively exploitable conditions can influence action through this pathway only when the actor detects and represents them as usable for obtaining an advantage. GET does not presently posit a specialized exploitability-detection mechanism; whether sensitivity to exploitable conditions reflects a distinct disposition or more general processes of learning, attention, and social cognition remains an empirical question.

Broader personality characteristics may influence several appraisal dimensions simultaneously. Antagonistic and aversive traits are associated with self-serving, manipulative, and non-cooperative behavior, whereas Honesty–Humility is inversely associated with tendencies toward exploitation, manipulation, and rule-breaking for personal gain (Ashton and Lee, 2007; Moshagen et al., 2018; Muris et al., 2017; Thielmann et al., 2020). GET treats such traits as distal predictors rather than explanatory endpoints. Their theoretical value increases when their association with exploitative behavior can be traced to more specific pathways, such as higher expected actor benefit, lower anticipated self-cost, greater perceived appropriability or, when exploitation is expected to be worse for the target than available alternatives, lower target-welfare weighting.

This calibration perspective reconciles stable individual differences with within-person variation. Actors may possess enduring tendencies in reward sensitivity, empathy, temporal valuation, or personality while evaluating comparable opportunities differently across relationships and situations. Relationship closeness can change target-welfare weighting; incentives can alter expected actor benefit; accountability can change anticipated self-cost; and new information can alter perceived appropriability. GET therefore predicts neither a fixed exploitative personality nor a purely situational process. Stable individual characteristics can bias appraisal systematically while leaving substantial scope for contextual recalibration.

### 3.4 Social-contextual conditions and the ecology of constraint

The second major source of calibration is the relational and social environment. GET uses the term constraint ecology to refer to the configuration of relational, reputational, normative, and institutional conditions that alter objective exploitability, exploitation-relevant appraisal, and the expected consequences of exploitative action. Constraint ecology does not denote a single quantity of external control. Different contextual conditions can affect different components of the causal architecture, combine with one another, or partially substitute for one another in limiting or facilitating exploitation.

Some contextual conditions primarily influence anticipated self-cost. Monitoring and transparency can increase the expected likelihood of detection, whereas accountability and credible enforcement can increase anticipated material, reputational, or institutional consequences. Conversely, anonymity, diffuse responsibility, or inconsistent enforcement may reduce some of these anticipated costs. Research on cooperation and institutional governance demonstrates that monitoring, punishment, accountability, and enforcement can substantially alter incentives surrounding cooperative and non-cooperative behavior (Fehr and Gächter, 2002; Ostrom, 1990; Tyler, 2006). Within GET, these conditions matter not because weak constraint defines exploitation, but because they can recalibrate the expected consequences of acting on an exploitable situation.

Other contextual conditions primarily affect objective exploitability and perceived appropriability. Dependency, limited exit options, informational inequalities, control over valued resources, and institutional authority can create or increase objective exploitability by making position-dependent advantage available. Conversely, access to alternatives, bargaining capacity, independent oversight, or credible resistance can reduce objective exploitability and, when recognized by the actor, perceived appropriability. Power-dependence approaches similarly emphasize that dependence varies with the value of resources controlled by another party and the availability of alternatives (Emerson, 1962). These conditions do not merely constrain exploitation after an opportunity has arisen; they can help constitute the relational environment from which exploitability itself emerges. Nevertheless, dependency or inequality does not establish exploitation, because objectively exploitable conditions may remain unused.

Reputational visibility and repeated interaction can operate through related pathways. Behavior that is observable to others can affect access to future partners, status, trust, or valued relationships, thereby increasing the potential longer-term costs of exploitative action. Repeated interaction can similarly connect present behavior to future cooperation or retaliation, and extensive work on repeated social interaction shows how future consequences can support cooperation (Axelrod and Hamilton, 1981; Nowak and Sigmund, 2005). Yet repeated interaction is not intrinsically protective. Stable relationships can also generate dependency, informational advantages, or accumulated control. What matters for GET is how the relational structure changes objective exploitability and recalibrates expected actor benefit, anticipated self-cost, perceived appropriability, and target-welfare weighting.

Normative environments can influence several appraisal pathways simultaneously. Norms can make exploitative conduct reputationally costly, increase expectations of sanction, or become internalized as standards that generate psychological self-cost. Research on social norms and cooperation shows that normative expectations and sanctioning institutions can substantially shape social behavior (Fehr and Gächter, 2002; Henrich et al., 2006; Ostrom, 1990). Norms can also normalize unequal practices, provide justifications for them, or weaken the perceived legitimacy of resistance (Jost, 2020). Norms are therefore neither inherently inhibitory nor definitional of exploitation. Their relevance within GET lies in how they alter anticipated consequences, social expectations, and interpretations of the interaction.

The term constraint ecology emphasizes that contextual mechanisms operate configurationally. Formal sanctions may have little influence without effective monitoring; reputational incentives may be weakened when behavior is difficult to observe or connect to a stable identity; strong relational dependence may limit the practical effects of otherwise substantial formal protections; and access to exit may reduce vulnerability even where institutional oversight is limited. Institutional and cooperation research similarly indicates that behavioral outcomes depend on configurations of rules, monitoring, enforcement, and incentives rather than on the nominal presence of any single regulatory mechanism (North, 1990; Ostrom, 1990). Apparently similar regulatory environments can therefore produce different effects because their elements combine differently and operate through different points in the causal architecture. GET consequently replaces the earlier notion of generally “weak constraints” with the more specific question of which contextual conditions alter which components of objective exploitability and exploitation-relevant appraisal.

Contextual influences should also not be regarded as permanently exogenous. Monitoring, dependency, normative expectations, or institutional enforcement may function as antecedent conditions in one interaction while themselves having been shaped by earlier behavior. Institutional approaches likewise emphasize that rules and enforcement structures both shape behavior and are maintained or transformed through repeated social processes (North, 1990; Ostrom, 1990). Their causal status therefore depends on the temporal point under examination. This feature becomes central once outcomes are incorporated into the recursive component of GET.

### 3.5 From appraisal to exploitative action and outcome

Exploitation-relevant appraisal organizes information relevant to action but does not determine behavior mechanically. An actor may expect substantial benefit, anticipate little personal cost, and perceive a highly appropriable opportunity while nevertheless refraining, whereas exploitation may occur under less favorable conditions when expected gains are especially valuable or consequences for the target receive little weight. GET therefore assumes a probabilistic relation between appraisal and behavior rather than a universal behavioral threshold.

Recognition of exploitability should likewise not be equated with exploitative motivation. An actor who identifies another party as exploitable may pursue the advantage, refrain from doing so, or act in ways that reduce the target’s vulnerability. GET is principally concerned with the first of these transitions, not with providing a general theory of protective behavior. The availability of alternative responses nevertheless demonstrates why objective or perceived exploitability cannot itself serve as a proxy for exploitation.

When exploitation is pursued, exploitative action refers to goal-directed behavior organized around obtaining self-benefit through a position-dependent advantage arising from a feature of another party’s relational position. Different behavioral mechanisms can implement this strategy. Deception can create or preserve informational advantage; coercion can restrict another party’s effective choices; cheating can circumvent rules or cooperative expectations; and manipulation, strategic withholding of information, cost shifting, or appropriation can provide other routes to advantage. These forms are not components of GET or alternative definitions of exploitation. They are behavioral means that become exploitative when they instantiate the relational structure defined in Section 2.

Exploitative action must be distinguished from exploitative outcome. An actor can attempt exploitation and fail because the target resists, third parties intervene, institutional protections operate, circumstances change, or the actor misjudges the situation. A successful exploitative action, by contrast, realizes the intended advantage through the target’s position. This distinction separates goal-directed action from realized consequence and allows the determinants of exploitative attempts to be studied separately from the conditions governing their success.

It also makes appraisal accuracy empirically relevant. Objective exploitability and its psychological representation can diverge. Actors may fail to recognize exploitable conditions, correctly identify them but misjudge their likely benefits or costs, or perceive an opportunity as appropriable when the relational conditions do not in fact support the intended advantage. Objective conditions, appraisal, behavioral choice, and outcome can therefore in principle be measured independently rather than inferred retrospectively from one another.

GET deliberately leaves the mathematical form of these transitions unspecified. The appraisal dimensions need not combine additively or multiplicatively, and no universal threshold or nonlinearity is assumed. Such functional forms may emerge in particular contexts, but they should be derived and tested empirically rather than built into the general theory. The general causal claim is more limited: relational and contextual conditions influence exploitative behavior partly through exploitation-relevant appraisal, and the relation between appraisal and action remains open to empirical specification.

Action and outcome also generate information and consequences that can alter subsequent interactions. Their role therefore extends beyond the immediate episode and leads to the recursive component of GET.

### 3.6 Feedback across time and levels

GET treats exploitation as a recursive rather than purely unidirectional process. Outcomes can modify both subsequent appraisal and the relational or social-contextual conditions under which later interactions occur. A variable that functions as an antecedent at one point in time can therefore become the consequence of earlier interactions and an antecedent again in subsequent ones.

At the actor level, outcomes can recalibrate subsequent exploitation-relevant appraisal. Successful exploitation may increase expected actor benefit or perceived appropriability in similar situations, whereas failure, resistance, detection, or sanction may reduce perceived appropriability or increase anticipated self-cost. Such updating is consistent with the broader principle that the consequences of social interaction shape expectations and subsequent behavioral decisions across repeated encounters (Axelrod and Hamilton, 1981; Nowak and Sigmund, 2005). Outcomes can also alter target-welfare weighting, for example when repeated interaction increases relational value or when new information changes the weight assigned to another person’s welfare (Delton and Robertson, 2016; Sell et al., 2009). Feedback at this level therefore provides a mechanism through which experience can produce within-person change without requiring a change in the underlying definition of exploitation.

At the relational level, exploitative action and its consequences can alter dependency, trust, information, bargaining positions, exit options, and capacities for resistance. Successful exploitation may deepen dependency or reveal additional opportunities for advantage-taking, whereas resistance or withdrawal may reduce objective exploitability in subsequent interactions. Relational feedback can therefore change the conditions under which later appraisal and action occur.

At the social and institutional level, repeated behavior can influence expectations regarding what is normal, tolerated, and enforceable, while institutional responses can modify monitoring, accountability, sanctions, and opportunities for resistance. Institutional theories have long emphasized that rules and enforcement structures shape behavior while themselves evolving through social interaction and collective processes (North, 1990; Ostrom, 1990). In GET, this means that constraint ecologies can be reproduced or transformed by the behavior they regulate. Practices that repeatedly generate advantages without effective challenge may become embedded or normalized, whereas collective resistance, enforcement, regulation, or institutional reform may reduce subsequent exploitability. These changes can reshape the constraint ecology and objective exploitability faced by actors in subsequent interactions, while also altering how the expected benefits and costs of exploitative action are appraised.

These processes permit cross-level amplification as well as attenuation. Repeated success without sanction could simultaneously lower an actor’s anticipated self-cost, increase a target’s dependency, and weaken expectations of institutional intervention, jointly increasing the likelihood of subsequent exploitation. The reverse is equally possible: resistance can reduce expected actor benefit, sanctions can increase anticipated self-cost, improved alternatives can reduce dependency and objective exploitability, and institutional responses can reduce perceived appropriability or otherwise constrain available routes to advantage-taking. GET therefore does not assume that exploitative processes inevitably escalate.

Different forms of feedback are also likely to unfold on different timescales. Actor expectations may change after a single salient outcome, whereas dependency structures, normative expectations, or institutional arrangements may require repeated interactions or coordinated action to change. Timescale is therefore treated as a property of particular causal processes rather than as a separate universal mechanism.

The recursive architecture can be represented minimally as follows: relational and social-contextual conditions at time *t* contribute to objective exploitability and exploitation-relevant appraisal; appraisal shapes behavioral response; behavioral responses generate outcomes; and those outcomes modify actor-level, relational, or institutional conditions relevant to interactions at *t* + 1. This notation specifies temporal ordering without implying a particular mathematical feedback model or identical rates of change across levels. In this way, GET links psychological and social-institutional explanations without reducing either to the other: social conditions shape behavior partly through appraisal, while repeated behavior can in turn alter the conditions under which subsequent appraisal occurs.

## 4 Contextual instantiations of GET

### 4.1 Interpersonal exploitation

Interpersonal relationships provide a direct setting in which the relational architecture of exploitation can be observed. Friends, romantic partners, family members, coworkers, and other interaction partners often differ in access to information, resources, alternatives, social support, or the capacity to impose or resist costs. Such differences can create objective exploitability without constituting exploitation themselves. Their significance depends on whether they provide one party with a usable route to position-dependent advantage and on how that possibility is appraised.

Dependency illustrates this distinction particularly clearly. Power-dependence theory proposes that an actor’s power in a relationship derives partly from the other’s dependence on outcomes the actor controls and from the availability of alternatives to that relationship (Emerson, 1962). Research on close relationships similarly identifies the quality of alternatives and investments as important bases of dependence and commitment (Rusbult et al., 1998). Dependence on another for resources, emotional support, access, status, or continuation of a valued relationship may therefore increase objective exploitability by raising the costs of resistance or exit. Yet dependency remains neither necessary nor sufficient for exploitation: it becomes relevant when it provides a route through which the actor can obtain a position-dependent advantage.

Trust can create a different and more ambivalent route to interpersonal exploitability. Trust involves willingness to accept vulnerability to another party under conditions in which that party’s actions matter for one’s outcomes (Mayer et al., 1995). This vulnerability can facilitate cooperation while also creating informational, discretionary, or relational opportunities for advantage-taking. At the same time, trusting and committed relationships can inhibit exploitation through anticipated relationship loss and, when exploitation is expected to worsen the partner’s welfare relative to available alternatives, through stronger target-welfare weighting. Dependency and trust can therefore affect both the availability of an exploitative opportunity and its psychological attractiveness, helping explain why comparable relational conditions need not produce comparable behavior.

Interpersonal exploitation also illustrates the recursive character of GET. Repeated cost shifting or strategic use of dependency may reduce a target’s alternatives or resources and thereby increase subsequent objective exploitability, whereas detection may reduce trust, encourage exit, increase vigilance, or mobilize support, making subsequent exploitation more difficult. At the actor level, success without resistance may lower anticipated self-cost or increase perceived appropriability, whereas confrontation or relationship loss may recalibrate these appraisals in the opposite direction. Interpersonal exploitation can thus generate either amplification or attenuation as outcomes modify the actor, the target, and the relationship.

### 4.2 Institutional and organizational exploitation

Institutional and organizational settings are especially important for GET because exploitable conditions can be generated and maintained by roles, rules, and resource distributions extending beyond any single interpersonal relationship. Organizations allocate authority, control over valued resources, access to information, and opportunities for exit in systematically unequal ways. Research on workplace power and abusive supervision illustrates the behavioral significance of these dependencies: supervisory conduct and subordinate responses vary partly with employees’ mobility and dependence on the employment relationship (Tepper, 2000; Tepper et al., 2009). Such arrangements can create objective exploitability without constituting exploitation themselves; they become relevant when an institutional position enables an actor to obtain a position-dependent advantage from a feature of another party’s relational position.

Control over valued resources and alternatives is one important source of institutional exploitability. Organizational actors may control employment, income, promotion, information, scheduling, access to services, or other outcomes on which less powerful parties depend. Power-dependence theory predicts greater power for actors who control valued outcomes when alternatives are limited (Emerson, 1962), while organizational research shows that job mobility can modify employees’ responses to abusive authority (Tepper, 2000; Tepper et al., 2009). Costly exit and dependency can therefore increase objective exploitability, while formal authority may increase perceived appropriability by making compliance easier to obtain. Limited oversight or diffuse responsibility may simultaneously reduce anticipated self-cost.

The surrounding constraint ecology determines how these institutional opportunities are translated into expected consequences. Monitoring, transparent procedures, credible accountability, and enforceable sanctions can increase anticipated self-cost, but formal protections may be ineffective when enforcement is weak or their use imposes substantial personal costs. Research on whistleblowing, for example, indicates that organizational context, retaliation, and perceived effectiveness influence whether wrongdoing is reported (Mesmer-Magnus and Viswesvaran, 2005; Near and Miceli, 1995). Conversely, professional norms, peer monitoring, reputational consequences, and repeated interaction can constrain conduct even where formal regulation is limited. Institutional constraints are therefore configurational rather than reducible to the nominal presence of rules or oversight (North, 1990; Ostrom, 1990).

Organizational norms can further alter how available advantages are interpreted and evaluated. Behavioral-ethics research indicates that ethical conduct cannot be understood solely from individual characteristics because group and organizational environments shape responses to ethically relevant situations (Treviño et al., 2006). Practices that become normalized may weaken expectations of sanction or become easier to justify, whereas strong professional or organizational standards can increase reputational and psychological costs. Institutional legitimacy and exploitation nevertheless remain distinct: institutions can constrain exploitative conduct while also stabilizing conditions under which position-dependent advantage becomes routinely available.

Institutional exploitation also makes recursive feedback particularly visible. Repeated successful exploitation can redistribute resources, deepen dependency, establish organizational precedents, or reveal ineffective safeguards, thereby increasing subsequent objective exploitability. Resistance, increased oversight, enforcement, or institutional reform can produce the opposite trajectory by reducing exploitability, perceived appropriability, or increasing anticipated self-cost. This bidirectionality links individual action to institutional change without reducing either level to the other and is consistent with institutional approaches in which rules and enforcement structures both shape and are transformed through repeated interaction (North, 1990; Ostrom, 1990).

### 4.3 Digital and platform-mediated exploitation

Digital environments provide a distinctive setting for GET because technological systems can redistribute information, discretion, and control while altering both objective exploitability and the constraints surrounding its use. Platforms may control access to markets, audiences, information, or employment opportunities while shaping what users, workers, or consumers can observe about the rules governing those interactions. Research on platform labor and algorithmic management documents how digital systems can restructure worker autonomy, information access, monitoring, and managerial control (Kellogg et al., 2020; Rosenblat and Stark, 2016; Wood et al., 2019). These arrangements can create objective exploitability when technologically mediated control provides a route to position-dependent advantage.

Platform dependence is one important mechanism. Workers, sellers, creators, or users may depend on a platform for income, visibility, customers, information, or access to a valued network, while those controlling the platform may possess substantially greater capacity to alter rules, rankings, prices, or access. Research on digitally mediated work documents substantial informational and control asymmetries surrounding algorithmic management and the information available to workers about how their performance and opportunities are governed (Rosenblat and Stark, 2016; Wood et al., 2019). Limited alternatives and asymmetric control can therefore increase objective exploitability and perceived appropriability when they make another party’s position usable for obtaining additional advantage.

Information asymmetry provides a related mechanism. Digital systems can make the collection, aggregation, and analysis of information inexpensive for organizations while leaving users with comparatively limited knowledge of how their data are used or how algorithmic decisions affecting them are produced. Research on online manipulation and digital choice environments shows how information architectures and technological design can influence decisions while limiting users’ awareness of the processes shaping those choices (Susser et al., 2019). Such asymmetries can create objective exploitability when informational disadvantage enables one party to secure terms or appropriate value that would be unavailable, or less favorable, if that disadvantage were absent.

Digital environments also create distinctive constraint ecologies. Technological systems can increase monitoring and traceability while anonymity, pseudonymity, scale, fragmented responsibility, or jurisdictional complexity weaken other forms of accountability. Platform rules may provide formal protections while concentrating discretion over enforcement, and reputation systems may constrain some forms of misconduct while creating opportunities for strategic manipulation. Scalability, automation, personalization, data aggregation, and algorithmic opacity can likewise alter the costs and feasibility of advantage-taking or resistance. These technological affordances are not themselves exploitative; their relevance depends on whether they enable a position-dependent advantage within the relational structure in which they operate (Kellogg et al., 2020; Rosenblat and Stark, 2016).

Finally, digital systems can amplify feedback across levels. Successful advantage-taking may generate additional data, resources, market control, or dependency that increases subsequent objective exploitability, while technical design, terms of service, ranking systems, or automated procedures can preserve the consequences of earlier decisions across later interactions. Conversely, user resistance, regulatory intervention, transparency requirements, platform redesign, or improved alternatives can modify the constraint ecology and reduce subsequent exploitability. Digital environments are therefore distinctive not because they require a different exploitation architecture, but because technologically mediated control, information asymmetry, platform dependency, and rapidly modifiable constraints can create and transform its relational inputs at unusual scale and speed.

### 4.4 What varies across contexts—and what does not

The preceding examples show that the concrete sources of exploitability are context-specific. In interpersonal relationships, exploitability may emerge from dependency, trust, unequal alternatives, or access to personally valued resources; in organizations and institutions, from authority, role structure, resource control, exit costs, monitoring, or enforcement; and in digital environments, from information asymmetries, algorithmic control, platform dependency, or technological choice architecture. Contexts likewise differ in the consequences actors anticipate and in the behavioral means through which position-dependent advantage can be pursued. Relationship loss may be especially salient interpersonally, formal sanctions or career consequences organizationally, and informational advantage, automated control, or behavioral traceability digitally.

What remains invariant is the proposed explanatory architecture rather than its substantive inputs. Across contexts, conditions such as dependency, hierarchy, information asymmetry, limited alternatives, or platform control can create objective exploitability without themselves constituting exploitation. Objective exploitability must therefore be distinguished from exploitation-relevant appraisal—expected actor benefit, anticipated self-cost, perceived appropriability, and target-welfare weighting—and both must be distinguished from behavioral response and realized outcome. Feedback follows the same architectural principle: although its content and timescale vary, outcomes can modify actor-level appraisals, relational conditions, and broader institutional environments relevant to subsequent interactions.

GET’s claim to generality is therefore architectural rather than a claim of substantive uniformity. The theory does not predict that identical variables, behavioral strategies, or constraints will explain exploitation equally well across domains. It predicts that context-specific mechanisms can be organized within a common sequence linking relational conditions, objective exploitability, exploitation-relevant appraisal, behavioral response, outcome, and feedback. The empirical value of this claim depends on whether that organization generalizes across substantively different contexts while remaining more informative than domain-specific description alone. This provides the basis for positioning GET relative to existing explanatory frameworks and for deriving the comparative predictions developed in the following sections.

## 5 Positioning GET within existing frameworks

GET draws on several established traditions rather than replacing them. Evolutionary and ecological approaches explain why behavioral strategies vary with recurrent adaptive problems and environmental conditions (Del Giudice et al., 2015; Ellis et al., 2009; Frankenhuis and Nettle, 2020); personality models identify stable individual differences associated with antagonistic and self-serving behavior (Ashton and Lee, 2007; Moshagen et al., 2018); person–situation approaches explain why behavioral expression depends on both persons and contexts; and structural or institutional theories identify social arrangements that distribute power, resources, information, and opportunities unequally (North, 1990; Ostrom, 1990). Each addresses processes relevant to exploitation. The distinctive claim of GET is narrower: these processes can be connected within an exploitation-specific causal architecture that distinguishes objective exploitability from its psychological appraisal, separates appraisal from action and outcome, and incorporates feedback through which behavior can modify subsequent relational and institutional conditions.

### 5.1 Evolutionary and ecological approaches

Evolutionary approaches provide an important foundation for understanding why humans possess psychological systems capable of conditional cooperation, competition, deception, and strategic advantage-taking. Life history and related ecological-calibration perspectives further emphasize that developmental and current environments can influence risk taking, temporal orientation, resource allocation, and other behavioral strategies (Del Giudice et al., 2015; Ellis et al., 2009; Frankenhuis and Nettle, 2020). More specifically, evolutionary analyses of exploitation have proposed that recurrent opportunities to benefit from others could have selected for psychological mechanisms sensitive to exploitable vulnerabilities and countermechanisms for detecting or resisting exploitation (Buss and Duntley, 2008). GET is compatible with this functional reasoning but does not require a specialized adaptation for exploitation or treat ecological calibration as a sufficient proximal explanation of exploitative action.

The distinction lies primarily in explanatory level and timescale. Evolutionary and developmental approaches can explain why individuals differ in psychological characteristics relevant to exploitation and why those characteristics may be calibrated by environmental experience. GET instead focuses on the proximal transition from a particular relational configuration to a behavioral response. An actor with relatively stable reward sensitivity, temporal preferences, or other-regarding tendencies may exploit in one situation and refrain in another because expected actor benefit, anticipated self-cost, perceived appropriability, or target-welfare weighting changes across relationships and contexts. Stable individual differences remain relevant, but they enter the model as calibrators of appraisal rather than as exploitative strategies in themselves.

This distinction generates an important complementarity. Evolutionary approaches can address the origins and functional organization of psychological systems relevant to exploitation, whereas GET specifies how those systems interact with current relational and social-contextual information in producing particular actions. GET therefore does not compete with evolutionary explanation; it provides a proximal architecture into which evolutionary hypotheses about the origins and calibration of relevant psychological mechanisms can be inserted.

### 5.2 Aversive personality and individual-difference models

Aversive personality models provide a second important perspective on exploitation. Constructs such as the Dark Triad and the Dark Factor of Personality capture relatively stable tendencies toward antagonism, manipulation, callousness, self-interest, and behavior that benefits the self at others’ expense (Jonason and Webster, 2010; Moshagen et al., 2018; Paulhus and Williams, 2002). Conversely, Honesty–Humility within the HEXACO framework captures individual differences in sincerity, fairness, greed avoidance, and modesty and is negatively associated with manipulative and exploitative tendencies (Ashton and Lee, 2007; Lee and Ashton, 2005). These models therefore identify dispositional characteristics that should be relevant to exploitation, but they do not by themselves specify when a particular relational situation will produce exploitative action.

GET locates the contribution of such traits within exploitation-relevant appraisal. Aversive traits may influence the subjective value of self-benefit, the weight assigned to another party’s welfare, the anticipated psychological costs of advantage-taking, or the readiness with which exploitable conditions are recognized and interpreted as usable. Honesty–Humility may affect partly complementary processes by increasing the weight of fairness-related considerations or reducing the attractiveness of gains obtained through manipulation or advantage-taking. The point is not that each personality dimension maps uniquely onto one appraisal component. Rather, GET provides a framework for testing which appraisal pathways mediate associations between broader dispositional characteristics and exploitative behavior.

This mapping also explains why trait measures should not be expected to determine behavior uniformly across contexts. An actor with a relatively low dispositional propensity toward antagonistic or self-serving behavior may nevertheless exploit when expected actor benefit is sufficiently valuable, anticipated self-cost is low, and perceived appropriability is high; when exploitation is expected to leave the target worse off than alternatives, weak target-welfare weighting may further favor exploitation. Conversely, an actor with a strong antagonistic or self-serving propensity may refrain when anticipated self-cost is high, perceived appropriability is low, or strong target-welfare weighting favors a non-exploitative response with a better expected target outcome.

GET consequently treats personality as an important but incomplete level of explanation. Trait models describe relatively stable differences between actors; GET asks through which proximal psychological dimensions those differences become relevant in a particular relational situation. This distinction generates testable mediation and moderation questions that trait models alone do not require. If aversive personality predicts exploitative action, GET predicts that at least part of this association should be traceable to exploitation-relevant appraisal, while contextual conditions should alter the behavioral expression of the underlying disposition.

### 5.3 Person–situation and interactionist approaches

GET is perhaps most directly aligned with person–situation approaches, which reject explanations of behavior based solely on either stable dispositions or external situations. Contemporary interactionist research shows that behavior reflects both person characteristics and psychologically meaningful situation characteristics and that trait expression varies substantially across situations (Fleeson, 2001; Funder, 2006; Rauthmann et al., 2015). This general premise is fully consistent with GET’s claim that neither exploitative dispositions nor exploitable environments determine behavior independently.

GET’s proposed contribution is to specify the content of this interaction for exploitation. “Person × situation” is a general explanatory principle, but it does not by itself identify which features of situations are exploitation-relevant, how actors represent them, or through which proximal psychological dimensions they influence action. GET proposes that relational and contextual conditions create or modify objective exploitability and that their behavioral significance is mediated through exploitation-relevant appraisal, including expected actor benefit, anticipated self-cost, perceived appropriability, and target-welfare weighting. Individual differences and contextual conditions can affect the same appraisal dimensions, providing a common psychological currency through which person and situation jointly shape behavior.

The recursive component adds a second distinction. In many person–situation formulations, the situation is treated as the current context in which behavior is expressed. GET additionally makes the consequences of behavior part of the subsequent causal environment. Exploitative outcomes can change dependency, trust, available resources, anticipated sanctions, institutional practices, or other conditions relevant to later interactions. Person and environment are therefore connected not only through contemporaneous interaction but through feedback across time. This combination of a structured proximal mechanism and cross-level feedback distinguishes GET from a generic invocation of person–situation interaction.

### 5.4 Structural and institutional accounts

Structural and institutional approaches begin from a different explanatory direction. Research on power, social hierarchy, political and economic institutions, and institutional governance shows how access to resources, authority, information, alternatives, and enforcement can be distributed asymmetrically and how these arrangements shape behavior and outcomes (Acemoglu and Robinson, 2012; North, 1990; Ostrom, 1990; Sidanius and Pratto, 1999). Such approaches are indispensable for explaining why some relational positions provide greater opportunities for advantage-taking and why these opportunities may become stable or recurrent.

GET does not attempt to replace structural explanation with psychological explanation. Instead, it distinguishes structural conditions from the proximal processes through which actors respond to them. Institutional authority, dependency, informational advantage, or weak enforcement can increase objective exploitability, alter anticipated self-cost, or change perceived appropriability, but none determines exploitative action by itself. Actors must detect and represent relevant features of the situation, and comparable structural positions can therefore produce different behavior across individuals or even within the same individual at different times.

The reciprocal direction is equally important. Structural conditions are not always fixed antecedents. Repeated actions can redistribute resources, alter dependency, change expectations of enforcement, normalize practices, or provoke institutional responses. GET therefore connects micro-level behavior to macro-level conditions without claiming that either level can be reduced to the other. Structural theories identify important sources and consequences of exploitability; GET adds a proximal psychological pathway linking those conditions to action and a recursive pathway through which accumulated actions can contribute to subsequent structural change.

The relationships between GET and these adjacent explanatory traditions are summarized in Table 2. Rather than ranking the frameworks by explanatory breadth, the comparison identifies their primary explanatory targets and the roles they assign to individual differences, current relational and contextual conditions, proximal psychological processes, cross-level integration, and recursive feedback. This comparison also clarifies that GET’s proposed contribution lies not in any one of these elements individually, but in their organization around the causal explanation of exploitative relational action.

**Table 2:** Positioning GET relative to adjacent explanatory frameworks.

Framework	Primary explanatory target	Individual differences	Current relational/contextual conditions	Proximal psychological mechanism	Cross-level integration	Recursive feedback
Evolutionary/ecological approaches	Origins and calibration of behavioral strategies	Important	Important as ecological inputs	Varies across models	Possible	Varies
Aversive personality models	Stable individual differences in antagonistic/self-serving behavior	Central	Usually secondary	Trait-linked processes vary across models	Limited	Not primary
Person–situation approaches	Context-dependent behavioral expression	Central	Central	Psychological interpretation of situations is important	Usually centered on person–situation relations	Varies
Structural/institutional approaches	Distribution of power, resources, opportunities, and constraints	Usually secondary	Central	Often not the primary explanatory level	Central	Often important
GET	Exploitative relational action and outcome	Calibrate appraisal	Create or modify objective exploitability and calibrate appraisal	Exploitation-relevant appraisal	Explicit psychological ↔ relational/institutional linkage	Explicit component of the theory

### 5.5 The incremental explanatory role of GET

The contribution of GET does not depend on claiming that objective exploitability, appraisal, personality, institutional constraint, or feedback is individually novel. Each has antecedents in established literatures. The theoretical claim concerns their organization around a specifically defined explanandum. GET separates the question of what constitutes exploitation from the question of what makes exploitative action more likely and then specifies a causal sequence linking relational conditions to objective exploitability, exploitation-relevant appraisal, behavioral response, outcome, and feedback.

These claims should not be interpreted as implying that appraisal, contextual sensitivity, concern for others, or feedback are unique to GET. More general social-cognitive and person–situation frameworks already accommodate psychologically mediated context effects and learning from previous interactions. For example, the Cognitive-Affective Processing System framework explains behavioral variability through situation-sensitive cognitive-affective units and person-specific patterns of responding (Mischel and Shoda, 1995), while social information-processing models specify sequential processes of encoding, interpretation, goal formation, response generation, and response evaluation (Crick and Dodge, 1994). Broader social-cognitive approaches likewise allow reciprocal influence between persons, behavior, and environments (Bandura, 1986). These frameworks therefore provide more demanding alternatives than models based solely on stable traits or objective situations.

GET’s incremental claim is correspondingly narrower. It proposes that, for exploitation specifically, relational conditions should be decomposable into objective exploitability and its psychological appraisal; that appraisal should be organized around expected actor benefit, anticipated self-cost, perceived appropriability, and target-welfare weighting; that these processes should predict specifically exploitative rather than merely self-interested or antisocial responses; and that outcomes can modify later exploitation through distinguishable actor-level, relational, and social-institutional feedback pathways. Evidence that context affects cognition, that cognition mediates behavior, or that previous outcomes influence later behavior would therefore be consistent with GET but would not discriminate it from these broader alternatives.

More discriminating support would require comparative tests of this exploitation-specific organization. GET would be favored when models preserving its distinctions among objective exploitability, appraisal, action, outcome, and level-specific feedback provide better explanatory or predictive performance than plausible alternatives that allow contextual appraisal and learning but do not require this particular architecture. Conversely, if broader social-cognitive models explain exploitative behavior equally well without these distinctions, or if the proposed GET-specific appraisal pathways fail to generalize across forms of position-dependent advantage, the incremental value of GET would be substantially reduced.

## 6 Empirical implications and distinctive predictions

Many empirical patterns discussed in relation to exploitation are already predicted by established theories. Power and dependency can shape opportunities for self-serving or abusive conduct (Emerson, 1962; Tepper et al., 2009), aversive personality traits are associated with antagonistic and manipulative behavior (Moshagen et al., 2018; Muris et al., 2017), monitoring and sanctioning can constrain misconduct or noncooperative behavior (Fehr and Gächter, 2002; Ostrom, 1990), and behavioral expression varies substantially across situations (Fleeson, 2001; Rauthmann et al., 2015). Such findings are relevant to GET, but they do not constitute distinctive tests of the theory. They are better treated as explanatory targets: established regularities that GET seeks to connect within a common causal architecture. Stronger tests require predictions that follow from the organization of that architecture rather than from any one of its constituent literatures.

The revised framework generates five empirical predictions derived from its causal architecture. These predictions differ in their discriminating power. Some test components that GET shares with broader social-cognitive or interactionist accounts, whereas others provide stronger tests of the exploitation-specific organization proposed here. Their evidential value therefore depends partly on whether they merely confirm processes accommodated by multiple theories or distinguish the particular pathways specified by GET.

### 6.1 Appraisal-mediation prediction

The most direct prediction of GET is that the effects of relational and social-contextual conditions on exploitative action should be explained partly by identifiable changes in exploitation-relevant appraisal. Manipulating or observing dependency, informational advantage, monitoring, accountability, access to alternatives, or other relevant conditions should therefore predict behavior through changes in one or more of four appraisal dimensions: expected actor benefit, anticipated self-cost, perceived appropriability, and target-welfare weighting.

This prediction is stronger than the claim that situations affect behavior. Consider an experimental manipulation that reduces a target’s ability to resist. GET predicts not merely an increase in exploitation but a more specific pathway: the manipulation should increase objective exploitability and, when detected by the actor, increase perceived appropriability. Similarly, increasing accountability should influence exploitation principally insofar as it changes anticipated self-cost rather than because “accountability” functions as an independent inhibitory force. Different contextual manipulations should therefore produce distinguishable psychological pathways.

The relevant test is not whether appraisal statistically mediates every contextual effect completely. Direct pathways, measurement error, omitted appraisals, and context-specific processes may remain. The stronger theoretical requirement is that appraisal should explain meaningful variance linking exploitation-relevant conditions to behavior and should improve explanation beyond distal individual differences and objective contextual characteristics alone. GET would be weakened if reliable changes in exploitative behavior produced by relational or contextual conditions occurred without corresponding changes in theoretically relevant appraisal, or if appraisal measures provided no incremental explanatory value once traits and objective conditions were known.

### 6.2 Functional-equivalence prediction

Because GET organizes antecedents according to the appraisal dimensions they affect, different objective conditions should sometimes produce similar behavioral consequences when they alter the same appraisal pathway. This functional-equivalence prediction follows from the architecture rather than from the surface characteristics of particular contexts.

For example, formal sanctions, reputational visibility, and the prospect of relationship loss are substantively different constraints. Yet all can increase anticipated self-cost. If their effects on exploitation operate principally through that appraisal dimension, then interventions producing comparable changes in anticipated self-cost should produce correspondingly similar changes in exploitative action, other relevant appraisal dimensions being held approximately constant. Likewise, informational superiority, target dependency, and control over alternatives may arise through different mechanisms but can converge on perceived appropriability when they make the target’s position appear more usable for obtaining an advantage.

This prediction permits both convergence and differentiation. Two interventions conventionally grouped together as “constraints” need not have equivalent effects if one primarily increases anticipated self-cost while another primarily reduces perceived appropriability. Conversely, conditions drawn from very different domains may prove functionally similar when they alter the same appraisal dimension. GET therefore predicts that classifying antecedents by their proximal appraisal effects should explain behavioral variation better than classifying them solely by their surface domain or conventional category.

The prediction would be weakened if contextual antecedents affected exploitative behavior in entirely idiosyncratic ways that could not be organized around the proposed appraisal dimensions, or if interventions producing comparable changes in a given appraisal dimension systematically produced unrelated behavioral consequences without identifiable moderating conditions.

### 6.3 Contextual-recalibration prediction

GET predicts systematic within-person variation in exploitative behavior because appraisal is sensitive to current relational and contextual information. Stable individual differences should therefore predict average tendencies without fixing behavior across situations. The same actor should become more or less likely to pursue exploitation when expected actor benefit, anticipated self-cost, perceived appropriability, or target-welfare weighting changes across contexts.

This prediction can be tested using repeated-measures experiments, experience-sampling designs, longitudinal interaction data, or multilevel studies in which the same individuals encounter systematically varying relational conditions. For example, increasing monitoring should raise anticipated self-cost within individuals; changing the target from a distant stranger to a valued relationship partner may increase target-welfare weighting; and reducing a target’s alternatives may increase perceived appropriability. GET predicts that corresponding within-person changes in exploitative behavior should be partly accounted for by within-person changes in these appraisal dimensions.

The critical implication is within-person contextual recalibration rather than a necessary statistical person × situation interaction. GET predicts that theoretically relevant changes in relational or social-contextual conditions should produce corresponding changes in one or more appraisal dimensions within the same actor, and that these appraisal changes should help account for subsequent behavioral variation. A contextual manipulation could therefore shift appraisal and behavior in a similar direction across actors and still support this prediction.

Person × situation interactions constitute a stronger, conditional hypothesis rather than a necessary consequence of GET. Such interactions are expected only when stable actor characteristics alter sensitivity to, interpretation of, or weighting of a particular contextual input. For example, individual differences in concern for others could moderate the extent to which information about target vulnerability changes target-welfare weighting, whereas differences in punishment sensitivity could moderate the effect of accountability on anticipated self-cost. These moderation hypotheses require independent specification of the relevant individual-difference mechanism and should not be inferred merely from the existence of contextual recalibration.

### 6.4 Divergent-response prediction

A particularly informative test of GET’s target-welfare component is that increased exploitability need not have a uniformly positive effect on exploitative behavior. Objective exploitability identifies a relational possibility, not a motivational direction. Its behavioral effect should depend on how the actor evaluates the available responses, including the expected welfare consequences of those responses for the target.

Target-welfare weighting therefore does not imply a general inhibitory effect on exploitation. Its behavioral implication depends on the target’s expected welfare under exploitation relative to the available alternatives. If exploiting an opportunity is expected to leave the target worse off than restraint or non-exploitation, greater target-welfare weighting should reduce the attractiveness of exploitation, other appraisal dimensions being held approximately constant. If, however, an exploitative transaction is expected to leave the target better off than non-interaction or restraint—as is possible in mutually beneficial exploitation—greater target-welfare weighting need not discourage exploitation and may favor it relative to an alternative that produces a worse outcome for the target. Where a protective response is available and is expected to produce still better target welfare, greater target-welfare weighting should instead increase the relative attractiveness of protection.

The divergent-response prediction is therefore inherently comparative. Consider a design in which objective exploitability is manipulated while actors can choose among exploitative, neutral or restrained, and protective responses, and the expected consequences of these responses for the target are independently varied. GET predicts that the effect of target-welfare weighting on behavioral choice should depend on these relative target outcomes. Greater target-welfare weighting should shift choice away from exploitation when exploitation is comparatively worse for the target, but not when exploitation provides the target with a better expected outcome than restraint. Protection should become increasingly attractive when it provides the best available target outcome. The direction of the association between target-welfare weighting and exploitation therefore has no fixed sign independently of the target-welfare contrast among available responses.

This prediction is stronger than the claim that empathy or prosociality reduces exploitation. Information indicating that another party is vulnerable or dependent can simultaneously signal an opportunity for position-dependent advantage and information relevant to the target’s welfare. The behavioral consequence depends not only on how strongly the actor weights the target’s welfare but also on what the actor expects exploitation, restraint, and any available protective response to mean for that welfare. Target-welfare weighting should therefore interact with expected target-outcome differences rather than operate as a context-independent brake on exploitative action.

Protective behavior is not itself an explanandum of GET, and the theory does not require protection to occur. Its theoretical importance here is diagnostic: including multiple response options and manipulating their expected consequences permits a stronger test of whether exploitability is being incorrectly equated with exploitative motivation. GET would be challenged if target-welfare weighting predicted behavior independently of expected target-outcome differences, or if experimentally varying the target’s relative welfare under exploitation, restraint, and protection failed to alter the behavioral effect of target-welfare weighting.

### 6.5 Recursive-feedback prediction

The feedback architecture generates an additional prediction for repeated interactions. Outcomes at one time point should modify conditions relevant to exploitation at subsequent time points. Successful exploitation without meaningful resistance or sanction may increase expected actor benefit or perceived appropriability, reduce anticipated self-cost, or alter relational conditions in ways that increase subsequent objective exploitability. Detection, punishment, target resistance, improved alternatives, or institutional intervention may instead increase anticipated self-cost, reduce perceived appropriability, or reduce objective exploitability, producing attenuation.

The distinctive implication is that repeated exploitation should not be modeled solely as the recurrence of a stable individual tendency. Earlier outcomes should predict later behavior partly through measurable changes in appraisal and relational or institutional conditions. Longitudinal and repeated-interaction designs could therefore test sequences in which exploitation at time *t* predicts changes in dependency, anticipated self-cost, perceived appropriability, or other relevant relational and appraisal conditions at time *t* + 1, which subsequently predict later behavior.

GET does not predict that feedback is necessarily self-reinforcing. Amplification and attenuation are both possible, and different components may change at different rates. The relevant prediction is temporal dependence: previous outcomes should systematically alter at least some of the conditions or appraisals governing later exploitative action. A purely static account in which prior outcomes add no explanatory information beyond stable traits and contemporaneous conditions would provide little support for the recursive component of GET.

### 6.6 What would count against GET?

Taken together, these predictions specify several ways in which GET can fail. The framework would be weakened if exploitation-relevant appraisal did not explain meaningful links between relational conditions and exploitative action; if the proposed appraisal dimensions failed to organize effects across substantively different antecedents; if theoretically relevant contextual changes did not produce systematic within-person recalibration; or if objective exploitability translated directly and uniformly into behavior regardless of expected actor benefit, anticipated self-cost, perceived appropriability, and target-welfare weighting. The recursive component would receive little support if outcomes failed to alter subsequent appraisal or relational and institutional conditions in repeated interactions.

Individual failures would not necessarily falsify the entire framework. A particular appraisal dimension may prove unnecessary, some pathways may be domain-specific, and alternative psychological representations may outperform those proposed here. Such findings should lead to revision rather than being absorbed by redefining exploitation or adding *post hoc* conditions. The architecture is empirically useful only insofar as its proposed intermediate processes improve prediction and explanation relative to simpler alternatives.

The appropriate tests of GET are therefore comparative. Relevant alternatives include cognitive-affective person–situation models that permit context-sensitive psychological mediation (e.g., Mischel and Shoda, 1995), social information-processing models that explain behavior through encoding, interpretation, goal formation, and response evaluation (e.g., Crick and Dodge, 1994), and broader social-cognitive models in which persons, behavior, and environments influence one another recursively (Bandura, 1986). Each can accommodate some combination of appraisal, contextual variation, individual differences, and learning from previous outcomes. Evidence for these processes alone therefore cannot discriminate GET from such accounts.

Stronger tests should compare preregistered GET-derived pathway models with alternatives that permit these general processes but omit or reorganize GET’s exploitation-specific components. Results would favor GET if independently measured objective exploitability is systematically distinguished from perceived appropriability, if context-specific antecedents operate through the predicted appraisal dimensions to explain specifically exploitative action, if this organization generalizes across substantively different forms of position-dependent advantage, and if previous outcomes predict later behavior through the proposed actor-level, relational, and social-institutional feedback pathways. Results would favor a broader alternative if comparable explanatory and predictive performance were obtained without these distinctions, if different appraisal structures consistently outperformed the proposed four dimensions, or if feedback were adequately explained as generic learning without systematic changes in subsequent exploitability or exploitation-relevant appraisal. Shared predictions remain useful tests of individual components of GET, but they should not be interpreted as discriminating evidence for the framework as a whole.

## 7 Discussion

### 7.1 Theoretical implications of GET

The principal theoretical implication of GET is that exploitation can be treated as a distinct relational explanandum without reducing its explanation either to characteristics of exploitative actors or to unequal social conditions. By separating the definition of exploitation from its causal explanation, the framework allows dependency, informational advantage, institutional authority, personality, and contextual constraints to remain empirical predictors rather than criteria built into the phenomenon itself. Objective exploitability similarly identifies a relational possibility rather than an exploitative action, leaving open the psychologically important question of how actors represent and respond to that possibility.

This organization also specifies the sense in which GET is intended to be general. Its generality is architectural rather than substantive: interpersonal, organizational, and digital exploitation need not share identical antecedents, behavioral forms, or constraints if context-specific mechanisms can nevertheless be organized around distinguishable relations among objective exploitability, exploitation-relevant appraisal, behavioral response, outcome, and feedback. The framework thereby connects psychological and structural levels without requiring either to be reduced to the other. Social arrangements can create exploitable positions and calibrate appraisal, while repeated actions can alter dependency, resources, expectations, and institutional constraints relevant to subsequent interactions.

The broader theoretical implication is methodological as well as substantive: GET’s generality and incremental value should be treated as empirical questions rather than theoretical assumptions. Future development should therefore constrain or revise the architecture when empirical findings fail to support its distinctions and pathways, rather than expand the framework to absorb discrepant results.

### 7.2 Scope conditions and limitations

The breadth of exploitation as a social phenomenon makes explicit scope conditions particularly important for a general theory. GET is intended to explain how exploitative relational action can emerge from the interaction between actors and relational or social-contextual conditions; it is not intended to provide a comprehensive theory of every phenomenon associated with exploitation. Several boundaries follow from this aim.

First, GET is not a general normative theory of exploitation. The minimal relational definition developed in Section 2 deliberately avoids making norm violation, absence of consent, or a particular theory of fairness necessary for identifying exploitation. This allows the causal framework to address interactions that may be socially accepted, institutionally legitimate, or mutually beneficial while still exhibiting the relevant relational structure (Mayer, 2007; Wertheimer, 1999). The corresponding limitation is that GET does not independently determine when an exploitative interaction is morally impermissible, how severe an instance of exploitation is, or which distribution of benefits and burdens would constitute a fair alternative. These questions require additional normative principles. The separation is intentional: incorporating a particular account of fairness into the causal definition would make GET dependent on theoretical commitments that are not necessary for explaining why exploitative action occurs.

Second, the minimal definition does not eliminate all classification problems at the boundaries of exploitation. Determining whether an actor obtains a position-dependent advantage from a feature of another party’s relational position may be straightforward in some cases and difficult in others. Complex exchanges can contain multiple benefits and costs, causal dependencies may be indirect, and institutional arrangements can distribute agency across many actors. The distinction between negative externality and exploitation, for example, depends on whether features of the affected party’s position are part of the process through which the advantage is pursued or secured rather than merely consequences of an independently beneficial activity. GET provides a criterion for making this distinction, but it does not guarantee that every empirical case will permit an unambiguous classification. Developing reliable procedures for identifying and operationalizing position-dependent advantage across different relational settings remains an important methodological challenge.

Third, the proposed appraisal architecture is deliberately provisional and empirically revisable. Expected actor benefit, anticipated self-cost, perceived appropriability, and target-welfare weighting were selected because they capture distinct psychological questions that must plausibly be resolved for an exploitable relational condition to acquire behavioral significance. GET does not assume that these dimensions exhaust all relevant psychological representations or that each will be equally important across contexts. Empirical work may reveal that some dimensions should be subdivided, that others covary too strongly to warrant separation, or that additional appraisal processes improve explanatory power. Such findings would require revision of the causal architecture rather than expansion of the definition of exploitation to preserve the model.

Relatedly, target-welfare weighting remains theoretically underspecified. Welfare trade-off models provide an established account of how actors may assign relative value to their own and others’ outcomes (Delton and Robertson, 2016; Sell et al., 2009), but GET does not equate target-welfare weighting with a welfare trade-off ratio or specify how empathy, relationship value, moral concern, kinship, reciprocity, or other social information combine to determine this weighting. The present framework requires only the more limited proposition that consequences for the target can enter appraisal independently of anticipated costs to the actor. The psychological representation and updating of target-welfare weighting therefore remain open empirical questions.

Fourth, GET currently specifies a causal architecture rather than a mathematical decision model. The theory does not assume that appraisal dimensions combine additively or multiplicatively, that exploitative action occurs when a fixed threshold is crossed, or that a common functional form applies across domains. This restraint avoids imposing mathematical assumptions for which there is presently insufficient evidence, but it also limits the precision of the framework. GET can identify variables and pathways that should be distinguished experimentally without yet specifying the quantitative function linking them. Formalization should therefore follow evidence about the measurement, independence, and interaction of appraisal dimensions rather than precede it.

A similar limitation applies to the recursive component. GET proposes that exploitative outcomes can modify subsequent appraisal, relational conditions, and social or institutional environments, but it does not yet provide a fully specified dynamic systems model of these feedback processes. Different feedback mechanisms are likely to operate on different timescales and may generate amplification, attenuation, equilibrium, or more complex trajectories. The current framework identifies temporal dependencies that should be tested but does not specify universal rates, lag structures, or conditions under which one trajectory must occur. Longitudinal and repeated-interaction evidence will be necessary before stronger dynamic claims are warranted.

Fifth, objective exploitability is necessarily context-sensitive in its operationalization. Dependency in a romantic relationship, limited exit options in an employment setting, informational disadvantage on a digital platform, and institutional control over resources are substantively different conditions. GET proposes that such conditions can perform a common theoretical function by making another party’s position usable for advantage-taking, but it does not imply that they should be measured by a single domain-general indicator. Establishing objective exploitability will therefore require domain-specific operationalization and, where possible, measurement independent of the actor’s appraisal. Such operationalization should identify the relevant relational feature and establish whether it provides a potential route to position-dependent advantage under the counterfactual criterion specified in Section 2.1, rather than inferring exploitability retrospectively from whether exploitation occurred.

Sixth, the contextual demonstrations developed in Section 4 should not be interpreted as an exhaustive taxonomy of exploitation. Interpersonal, institutional and organizational, and digital environments were selected because they instantiate the proposed architecture through substantially different concrete mechanisms. Other contexts may expose additional boundary conditions or reveal processes not adequately represented in the current framework. Cultural variation is particularly important. Norms, institutional arrangements, relationship expectations, and the meaning assigned to dependency, obligation, reciprocity, and legitimate advantage may differ across societies (Henrich et al., 2006). GET predicts that such variation can alter objective exploitability and appraisal without making prevailing norms definitional of exploitation, but the cross-cultural portability of the proposed architecture remains to be demonstrated rather than assumed.

Finally, GET is not intended as a general theory of cheating, deception, coercion, manipulation, or responses to vulnerability. These behaviors can become mechanisms through which exploitation is enacted, but each has causes and consequences extending well beyond exploitation. Similarly, the possibility that recognized exploitability may elicit restraint or protective action does not make protection a separate explanandum of GET. Such alternatives are theoretically relevant because they demonstrate that exploitability does not determine motivational direction. Their fuller explanation belongs to theories of cooperation, prosociality, morality, attachment, or social decision making.

These limitations place deliberate boundaries around the framework’s explanatory ambitions. GET should not expand by absorbing every phenomenon associated with unequal, harmful, deceptive, or self-interested interaction. Its development should instead depend on whether the relatively compact architecture proposed here survives empirical differentiation: whether exploitation can be identified independently of its hypothesized causes, objective exploitability can be distinguished from its appraisal, appraisal dimensions explain meaningful variation in behavior, and recursive pathways improve understanding of exploitation across time and levels. The immediate research priority is therefore not further conceptual expansion but increasingly stringent tests of these distinctions and causal pathways.

### 7.3 Future development and empirical priorities

The immediate priority for GET is empirical specification rather than further conceptual expansion. The predictions developed in Section 6 require research designs capable of separating relational conditions, psychological appraisal, behavioral response, and outcome rather than inferring one component from another. Several empirical priorities follow from this requirement.

First, the four proposed appraisal dimensions require reliable and discriminable measurement. Expected actor benefit, anticipated self-cost, perceived appropriability, and target-welfare weighting should be measured independently enough to determine whether they explain distinct variance in exploitative decisions and whether their structure is stable across contexts. Measurement development should also establish whether these dimensions are best represented as momentary appraisals, relatively stable individual tendencies, or—as GET anticipates—state-like evaluations systematically calibrated by more stable characteristics of actors.

Second, experimental research should manipulate objective exploitability independently of appraisal wherever possible. Designs could vary dependency, access to alternatives, informational advantage, capacity to resist, or institutional authority while separately measuring how actors represent those conditions. Such designs would permit direct tests of whether objectively comparable opportunities are perceived differently across individuals and whether experimentally induced changes in exploitability influence behavior through the appraisal pathways proposed by GET. They would also help distinguish failed detection of exploitability from deliberate restraint after an opportunity has been recognized.

Third, repeated-measures and multilevel designs are needed to test the framework’s emphasis on within-person contextual recalibration. Sampling the same individuals across systematically varying relational conditions would allow stable dispositional effects to be separated from context-sensitive changes in appraisal. Particularly informative studies would manipulate or observe theoretically specific contextual changes—such as monitoring, relationship closeness, target dependency, or reputational visibility—and test whether their behavioral effects correspond to predicted changes in particular appraisal dimensions.

Fourth, the architectural generality of GET requires comparative tests across substantively different contexts. The relevant question is not whether identical predictors explain interpersonal, organizational, and digital exploitation, but whether context-specific antecedents can be linked to behavior through comparable appraisal processes. Parallel experimental paradigms or coordinated multi-domain studies could therefore test whether different manipulations that alter anticipated self-cost or perceived appropriability generate functionally similar pathways despite their different substantive content. Failure to recover such common pathways would place meaningful limits on GET’s claim to architectural generality.

Finally, the recursive component requires longitudinal and repeated-interaction designs in which outcomes at one point can be linked to subsequent changes in actors, relationships, or institutions. Studies should examine whether successful exploitation, resistance, detection, or sanction changes later expectations of benefit and cost, alters dependency or available alternatives, or modifies relevant constraints. Such evidence would permit the present qualitative feedback architecture to be progressively replaced by more precise temporal and, where warranted, formal dynamic models.

These priorities suggest a cumulative research strategy: establish measurement, separate objective conditions from appraisal experimentally, test within-person recalibration, examine cross-context portability, and then model feedback across repeated interactions. The development of GET should depend on the results of these tests. Components that fail to provide incremental explanatory value should be revised or removed, while greater formalization should be introduced only where empirical evidence supports it. In this sense, the next stage of GET is less a matter of extending the theory’s conceptual reach than of progressively constraining it through measurement, comparison, and experimental and longitudinal evidence.

### 7.4 Conclusion

Exploitation is studied across disciplines that emphasize different parts of the phenomenon: individual dispositions, strategic behavior, relational inequalities, institutional arrangements, and broader social structures. GET seeks to connect these perspectives without collapsing them into a single level of explanation. Its starting point is a deliberately narrow relational definition of exploitation—self-benefit pursued or obtained through a position-dependent advantage enabled by a feature of another party’s relational position—separated from the causal conditions that make exploitative action more or less likely. This separation allows asymmetry, dependency, personality, constraint, and other familiar correlates of exploitation to remain empirical explanatory variables rather than becoming necessary features of the phenomenon by definition.

The resulting framework treats exploitation as a context-sensitive and recursive process. Relational and social-contextual conditions can create objective exploitability and calibrate how exploitable conditions are appraised. Exploitation-relevant appraisal, in turn, helps determine how actors respond to these possibilities. Exploitative actions and outcomes can subsequently alter actors, relationships, and institutional environments, thereby reshaping the conditions of later interactions. GET’s claim to generality lies in this causal architecture rather than in the assumption that exploitation has identical causes or behavioral expressions across contexts.

The value of GET will ultimately depend on whether this architecture improves explanation rather than merely redescribing established findings. Its central distinctions must remain empirically separable, its proposed mechanisms must account for meaningful variation beyond simpler trait-based or contextual models, and its cross-context and recursive claims must survive comparative, experimental, and longitudinal tests. A general theory of exploitation should become more constrained as evidence accumulates, not more encompassing. GET is intended to provide a framework within which that cumulative process can begin.

## Data Availability

The original contributions presented in the study are included in the article/supplementary material, further inquiries can be directed to the corresponding author.
